# Deciphering the Genomic Landscape of Oropharyngeal Squamous Cell Carcinoma: Distinct Mutation Patterns in Disease

**DOI:** 10.3390/life16020282

**Published:** 2026-02-06

**Authors:** Beau Hsia, Gabriel Bitar, Pedro S. Bonilla, Vinay D. Veluvolu, Nathan Tran, Saif Alshaka, Eli Oved, Bhavish Aubeelauck, Hassan Nur, Abubakar Tauseef, Vijay A. Patel, Aliasgher Khaku

**Affiliations:** 1 School of Medicine, Creighton University, Phoenix, AZ 85012, USAsaifalshaka@creighton.edu (S.A.); elioved@creighton.edu (E.O.); 2 College of Human Medicine, Michigan State University, Grand Rapids, MI 49503, USA; bonill32@msu.edu; 3 Carle Illinois College of Medicine, University of Illinois Urbana-Champaign, Urbana, IL 61801, USA; vinaydv2@illinois.edu; 4 School of Medicine, Texas Tech University Health Sciences Center, Lubbock, TX 79905, USA; nathan.s.tran@ttuhsc.edu; 5 CHI Health Clinic, Kearney, NE 68847, USA; bhavishaubeelauck@creighon.edu; 6 School of Medicine, Creighton University, Omaha, NE 68178, USA; hassannur@creighton.edu (H.N.); abubakartauseef@creighton.edu (A.T.); 7 Division of Pediatric Otolaryngology, Rady Children’s Hospital, San Diego, CA 92134, USA; vpatel2@rchsd.org; 8 H. Lee Moffitt Cancer Center and Research Institute, Tamp, FL 33612, USA; aliasgher.khaku@va.gov

**Keywords:** oropharyngeal squamous cell carcinoma, somatic mutations, genomic profiling, TP53, PIK3CA, AACR Project GENIE, targeted therapy

## Abstract

Objective: We aimed to characterize the somatic mutational landscape of oropharyngeal squamous cell carcinoma (OPSCC) and identify potential genomic drivers of tumor progression and therapeutic resistance using the AACR GENIE database. Study Design: Retrospective genomic analysis was employed. Setting: We used publicly available data from the American Association for Cancer Research (AACR) Project GENIE database accessed via cBioPortal. Methods: We analyzed 412 tumor samples from 401 patients diagnosed with OPSCC. Somatic mutations, clinical variables and tumor characteristics were extracted and analyzed. Statistical comparisons of mutation frequencies across gender and tumor stage (primary vs. metastatic) were conducted. Co-occurrence and mutual exclusivity analyses were performed to identify significant genomic patterns. Results: The most frequently mutated genes included *TP53* (30.1%), *PIK3CA* (26.0%), and *KMT2D* (21.6%). Gender-specific analyses suggested potential enrichment of *TP53* and *MET* mutations in females and of *ZNF750* in males. Distinct mutation patterns were observed between primary and metastatic tumors; primary tumors were enriched for mutations in *TP53* and *CDKN2A*, while metastatic lesions harbored unique alterations in genes like *CBLB* and *BUB1B*, suggesting pathways involved in immune evasion and chromosomal instability may drive disease progression. Co-occurrence was noted between *PIK3CA* and *FBXW7*, and mutual exclusivity between *TP53* and *CYLD*. Conclusions: This study identifies distinct genomic signatures in OPSCC subgroups, highlighting candidate biomarkers in pathways like PI3K/AKT signaling that warrant further investigation. Validating these markers in prospective trials is a critical next step to translate these findings into personalized therapeutic strategies for OPSCC patients.

## 1. Introduction

Oropharyngeal squamous cell carcinomas (OPSCCs) are malignant epithelial neoplasms arising from the mucosal surfaces of the oropharynx, including the base of the tongue, tonsils, soft palate, and pharyngeal walls. These tumors account for ~90% of oral cancers, with ~54,000 U.S. cases annually [1,2]. It is more common in males than females and has a unimodal age distribution at presentation, with most cases occurring within the fifth to seventh decades of life [3]. However, there is a rising prevalence among younger populations due to the increasing incidence of human papillomavirus (HPV)-associated cases. While HPV-positive cases are more frequent in middle-aged patients, HPV-negative cases predominate in older patients and are linked to traditional risk factors including tobacco and alcohol use [4,5]. Because of their malignant growth nature, OPSCCs present significant surgical challenges due to their proximity to critical neurovascular and muscular components such as the carotid artery, internal jugular vein, cranial nerves, sympathetic chain, and cervical plexus. At clinical presentation, patients often present with a sore throat, dysphagia, or most commonly a developing neck mass [6].

Surgical resection with neck dissection is the standard first-line treatment for OPSCC patients. Depending on the staging and additional adverse findings, adjuvant radiation or chemoradiation therapy may also be necessary [6]. Minimally invasive approaches like transoral robotic and laser microsurgery have largely replaced oral surgery [7,8]. For recurrent and metastatic cases, there is the emergence of immunotherapy with checkpoint inhibitors such as pembrolizumab and nivolumab and the standard of chemotherapy with cisplatin [8,9]. Beyond standard therapies, targeted agents such as EGFR inhibitors have shown limited efficacy as monotherapy, underscoring the need to identify more robust molecular targets [8]. The success of immune checkpoint inhibitors has revolutionized care for a subset of patients, yet many do not respond, highlighting a critical need to understand the genomic drivers of immune evasion and treatment resistance. Additionally, HPV status affects treatment outcomes as HPV-positive regimens of chemoradiotherapy have shown advantages due to a lower burden of co-morbidities with a more favorable prognosis compared to HPV-negative patients [9]. Despite these advances, recurrent OPSCC remains a significant challenge and systemic therapy remains a cornerstone for metastatic disease progression. An improved understanding of the tumor biology in OPSCC may lead to clinical trials introducing systemic and targeted therapies into the management of OPSCC.

The molecular understanding of OPSCC is advancing rapidly, with a critical distinction made between HPV-positive and HPV-negative disease, which are now considered distinct biological entities. HPV-positive tumors are often driven by viral oncoproteins, leading to functional inactivation of TP53 and RB, and are frequently associated with mutations in the PI3K/AKT/mTOR pathway, particularly in the *PIK3CA* gene [8]. Conversely, HPV-negative subtypes, linked to carcinogen exposure, typically harbor inactivating mutations in *TP53* and *CDKN2A* [5]. While these primary drivers are well established, a significant knowledge gap remains in understanding the full spectrum of somatic mutations that define tumor progression, metastatic spread, and therapeutic resistance across large, diverse patient populations.

Unlike previous studies that rely heavily on The Cancer Genome Atlas (TCGA) data, which is enriched for primary untreated tumors, this study utilizes the AACR GENIE database. This provides a ‘real-world’ view of OPSCC, including a significant proportion of metastatic and recurrent cases often underrepresented in earlier cohorts. By characterizing this diverse population, we aim to generate hypotheses regarding metastatic drivers and identify potential therapeutic vulnerabilities that may be missed in primary-only datasets.

## 2. Materials and Methods

This study was exempt from the Creighton University institutional review board approval as the database is deidentified and publicly available. The American Association for Cancer Research (AACR) Project Genomics Evidence Neoplasia Information Exchange (GENIE)^®^ database was accessed using the cBioPortal (v16.1-public) online software [5] on 22 July 2024, with clinical data dating back to 2017. Genomic sequencing information from 19 international cancer centers is compiled in the AACR GENIE^®^ database. Only a select number of cancer types include therapeutic response along with clinical outcomes data, but treatment regimens were not recorded for OPSCC. Additionally, each participating institution may use different pipelines from each other (and within the same institution). Participating institutions use either unbiased whole genomic/exome sequencing or targeted panels of up to 555 genes. It is important to note that HPV status for individual samples was not available within the queried AACR Project GENIE dataset, which represents a significant limitation acknowledged in this study.

We queried all patients with head and neck cancer and a pathologic diagnosis of OPSCC. The dataset included genomic data (e.g., somatic mutations), histological subtype, as well as clinical characteristics (e.g., race and age). Specific copy number alterations and structural variants were excluded from this analysis. Tumor mutational burden was calculated based on the number of detected somatic mutations. It should be noted that primary and metastatic samples in this cohort were distinct and not matched pairs from the same patients. Therefore, comparisons represent population-level differences rather than longitudinal evolutionary changes within individuals. Survival data was not available. Samples with missing data were excluded from the analysis to ensure the integrity of the results. Statistical analyses were conducted using R/R Studio (R Foundation for Statistical Computing, Boston, MA, USA), with significance set at *p*  <  0.05. Continuous variables were reported as means ± standard deviations (SD), and categorical variables were presented as frequencies and percentages. Differences between categorical variables were assessed using the chi-squared test. For comparisons of means between two groups, a two-sided *t*-test and nonparametric tests, such as the Mann–Whitney U test for non-normally distributed data, were applied. The Benjamini–Hochberg False Discovery Rate (FDR) correction was used to adjust for multiple comparisons.

## 3. Results

### 3.1. Oropharynx Squamous Cell Carcinoma Patient Demographics

The study included 401 patients with Oropharyngeal Squamous Cell Carcinoma (OPSCC), from whom 412 samples were collected. Of the patients, 399 (99.5%) were adults, 1 (0.25%) was pediatric, and 1 (0.25%) had no recorded age at the time of sample collection. The average age of the cohort was 61.8 ± 9.7 years. The group comprised 338 males (84.3%) and 63 females (15.7%). Of the 412 samples, 216 (52.4%) were from primary tumors, while 181 (43.9%) were from metastatic tumors. The tumor mutational burden across the cohort was 9.2 ± 13.8 mutations per megabase (mut/Mb). These results are outlined in Table 1.

### 3.2. Oropharynx Squamous Cell Carcinoma Top Somatic Mutations

**Figure 1:**
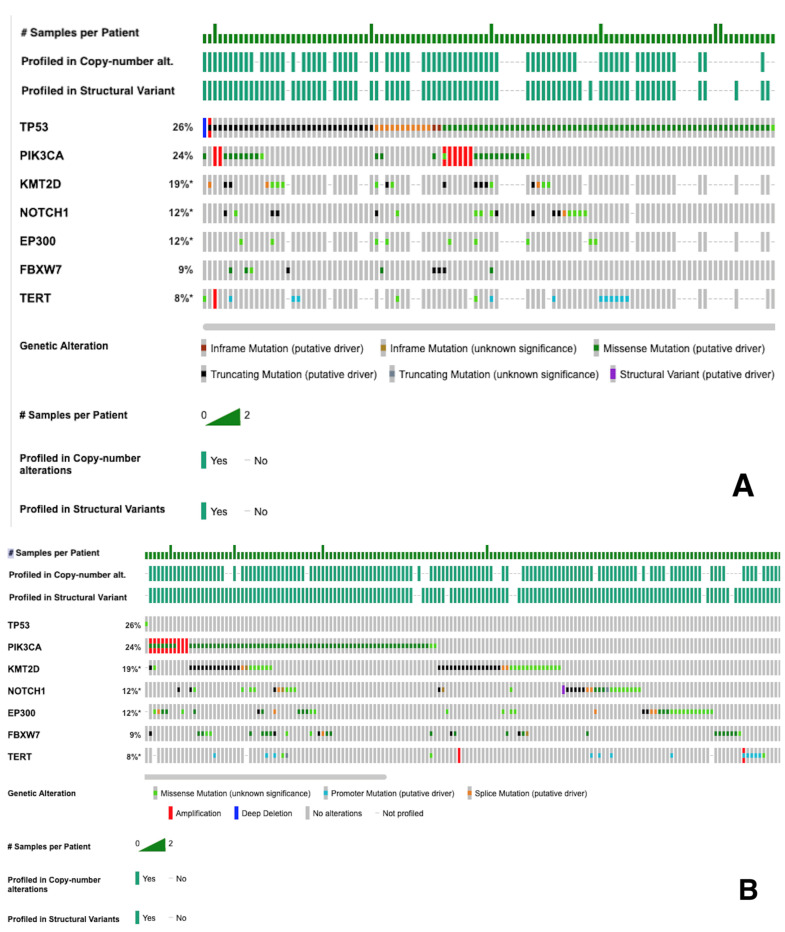
Oncoprint of recurrent mutations in OPSCC. (**A**,**B**) Single oncoprint split into two panels for readability. Alterations shown for genes present in ≥ 5 samples with VAF ≥ 5% and coverage ≥ 100×. * Not all samples were profiled.

The top mutations detected in the OPSCC cohort are summarized in Figure 1. The ten most common mutations were identified in the following genes: TP53 (n = 124; 30.1%), PIK3CA (n = 107; 26.0%), KMT2D (n = 89; 21.6%), NOTCH1 (n = 58; 14.1%), EP300 (n = 49; 11.9%), FBXW7 (n = 43; 10.4%), TERT (n = 36; 8.7%), FAT1 (n = 35; 8.5%), PRKDC (n = 31; 7.5%), and CYLD (n = 29; 7.0%).

Next, we explored the three most common mutations in more detail. For TP53, 124 mutations were identified in 108 samples, suggesting some patients had multiple mutations. Table 2 shows the types of mutations, with missense mutations being the most common. The three most frequently observed mutations were Y220C (n = 5; 4.0%), R175H (n = 5; 4.0%), and X187_splice (n = 4; 3.2%).

In PIK3CA, 107 mutations were identified in 88 patients, with all but one being missense mutations. The three most common mutations were E545K (n = 44; 41.1%), E542K (n = 26; 24.3%), and H1047R (n = 4; 3.7%). These distributions align with established HNSCC datasets (such as TCGA), where TP53 alterations are predominantly missense mutations affecting the DNA-binding domain, and PIK3CA mutations cluster in the helical (E542K, E545K) and kinase domains.

For KMT2D, 89 mutations were identified across 68 samples. Table 3 details the mutation types, with missense mutations being the most common, similar to TP53. Of the 89 mutations, 87 were unique to this gene, while the two repeated mutations were nonsense mutations: R2801* and R2471*. However, unlike TP53, a significant proportion of KMT2D alterations were nonsense or frameshift mutations (43.8% combined), consistent with its role as a tumor suppressor where loss-of-function is the primary oncogenic mechanism in head and neck cancers.

### 3.3. Gender-Specific Enrichment of Mutations in OPSCC

Analysis of gender-specific mutation patterns in the OPSCC cohort revealed notable differences in the prevalence of certain genetic alterations between males and females (Table 4). For example, mutations in MET, TP53, and TRAF3 showed significant *p*-values, suggesting enrichment in females, with higher percentages of female samples harboring these mutations compared to males. Similarly, ZNF750 and FGA mutations were enriched in males. While genes such as ZNF750 and MET showed nominal *p*-values (<0.05) suggesting potential gender-specific enrichment, these associations must be interpreted as exploratory trends rather than definitive drivers, as they may not maintain significance under strict FDR correction. Further investigation with larger cohorts or more refined statistical models can confirm gender-specific mutations play a significant role in the pathogenesis of OPSCC.

### 3.4. Mutation Patterns in Primary vs. Metastatic OPSCC

Comparative analysis of mutations between primary and metastatic OPSCC tumors revealed distinct enrichment patterns, highlighting potential differences in tumor biology and progression (Table 5). Several genes were significantly enriched in primary tumors, including TP53, which was mutated in 33.33% of primary samples compared to 18.23% of metastatic samples (Log2 Ratio = −0.87). Similarly, *CDKN2A*, *NOTCH2*, and *BRCA2* were more prevalent in primary tumors, with Log2 Ratios ranging from −1.19 to −1.9. *TSC1* and *TGFBR2* mutations were almost exclusive to primary tumors, observed in 4.39% and 6.49% of primary samples, respectively, but rarely in metastases (Log2 Ratios ≤ −2.92).

In contrast, several genes were enriched in metastatic tumors, suggesting roles in disease dissemination or progression. CBLB, BUB1B, EIF4A2, RAD54B, and PIK3CB mutations were exclusive to metastatic samples, exhibiting highly positive Log2 Ratios (>10). Results of this analysis can be seen in Table 5.

### 3.5. Co-Occurrence and Mutual Exclusivity of Mutations in OPSCC

We also examined the relationships between different mutations in the OPSCC cohort to identify patterns of co-occurrence (where mutations are found together) and mutual exclusivity (where mutations do not co-occur). Pairwise analysis revealed several significant co-occurrence and mutual exclusivity patterns among key genes (Table 6).

Several notable co-occurrence patterns were observed. For instance, mutations in PIK3CA (n = 107) and FBXW7 (n = 43) exhibited strong co-occurrence (Log2 odds ratio = 2.888, *p*-value < 0.001), meaning that tumors with mutations in PIK3CA were more likely to also harbor mutations in FBXW7. Similarly, mutations in KMT2D (n = 89) and FBXW7 (n = 43) were significantly associated (Log2 odds ratio = 2.554, *p*-value < 0.001). Co-occurrence was also observed between TP53 and TERT mutations (Log2 odds ratio = 2.043, *p*-value < 0.001), consistent with previous studies showing that these mutations often occur together, potentially influencing genomic stability and telomerase activity. Other notable co-occurrence pairs included TERT and FAT1 (Log2 odds ratio = 2.242, *p*-value = 0.001), NOTCH1 and PRKDC (Log2 odds ratio = 2.378, *p*-value = 0.002), and PIK3CA and KMT2D (Log2 odds ratio = 1.528, *p*-value < 0.001).

Mutual exclusivity was observed between TP53 and CYLD mutations (Log2 odds ratio = <−3, *p*-value < 0.001), indicating that tumors with TP53 mutations typically do not harbor CYLD mutations, and vice versa. This suggests that these mutations may influence separate cellular pathways, and the presence of one mutation may preclude the occurrence of the other. A similar pattern of mutual exclusivity was found between PIK3CA and CYLD mutations (Log2 odds ratio = <–3, *p*-value < 0.001).

These findings suggest that co-occurring mutations may act synergistically to enhance tumor growth or treatment resistance, while mutually exclusive mutations could activate alternative pathways in different subsets of tumors. These findings underscore the importance of considering not just individual mutations, but also how they interact with each other within the broader genetic network of OPSCC.

## 4. Discussion

In this study, we profiled the somatic mutational landscape of oropharynx squamous cell carcinoma (OPSCC) using a publicly available genomic database to identify secondary drivers of tumor progression and resistance, with the aim of informing novel therapeutic strategies and enhancing disease modeling. The mutational landscape reveals a complex interplay of genomic alterations that differ across patient subgroups, with significant complexity and distinct patterns of genetic alterations that have implications for tumor biology and potential therapeutic strategies. A critical lens for interpreting these findings is the absence of HPV status within the AACR GENIE OPSCC cohort. Given that HPV-positive and HPV-negative tumors represent distinct biological entities, the mutation frequencies reported here likely represent a composite of these two subtypes. Consequently, the patterns discussed below should be interpreted as characteristic of the heterogeneous real-world OPSCC population, rather than specific to viral etiology.

Our results complement existing TCGA data by providing a unique window into the metastatic landscape. While TCGA focuses largely on primary tumors, the inclusion of 181 metastatic samples in this cohort allowed for the identification of alterations in genes such as CBLB and BUB1B, which are rarely observed in primary disease and represent potential targets for advanced-stage therapy. The most frequently mutated genes in the OPSCC cohort were TP53 (30.1%), PIK3CA (26.0%), and KMT2D, with additional notable mutations in NOTCH1, EP300, and FBXW7. Among TP53 mutations, missense mutations predominated, with specific variants such as Y220C and R175H being prevalent, while PIK3CA mutations were also predominantly missense, with E545K and E542K as the most common alterations, and missense mutations similarly representing the majority of changes in KMT2D.

While high rates of PIK3CA alterations are frequently cited in the literature as a hallmark of HPV-positive disease, and TP53 mutations are often associated with HPV-negative status, we cannot definitively stratify our cohort based on these genomic surrogates alone. Therefore, the co-occurrence patterns observed here should be viewed as broad genomic signatures of OPSCC rather than specific viral-driven subtypes. The high prevalence of both TP53 and PIK3CA mutations suggests the cohort is likely a heterogeneous mix of HPV-negative and HPV-positive tumors, respectively, with TP53 mutation frequency aligning with rates typically seen in HPV-negative HNSCC and the high rate of PIK3CA alterations being a known hallmark of HPV-positive disease. This underlying heterogeneity is a critical context for interpreting our findings, particularly the patterns of mutual exclusivity and co-occurrence that may be driven by distinct HPV-related etiologies. Gender-specific differences in mutation prevalence were observed, but high q-values suggest these findings may not be statistically significant. Furthermore, a distinction was noted between primary and metastatic tumors, with mutations in genes like TP53 and CDKN2A enriched in primary tumors. Our analysis revealed a specific enrichment of CBLB, BUB1B, RAD54B, and PIK3CB mutations in metastatic samples. While these findings are hypothesis-generating, their biological functions align with metastatic progression. CBLB is an E3 ubiquitin ligase involved in immune regulation, and its mutation may facilitate immune evasion. BUB1B is a key component of the mitotic checkpoint; its alteration suggests chromosomal instability as a driver of metastatic competence. Notably, these alterations did not appear among the significant co-occurrence pairs with classical drivers like TP53 or PIK3CA (Table 6), suggesting they may represent distinct evolutionary trajectories or that larger cohorts are required to statistically power these specific gene-pair relationships. These alterations warrant further investigation as potential markers of metastatic risk.

Patterns of co-occurrence and mutual exclusivity provided additional insights into tumor biology. For instance, mutations in PIK3CA and FBXW7 showed significant co-occurrence, suggesting potential synergistic effects on tumor growth, whereas mutual exclusivity between TP53 and CYLD mutations indicates the activation of alternative pathways. These findings emphasize the necessity for further research to fully delineate the genomic alterations in OPSCC and to understand how these mutations influence tumor progression and response to therapy. Unraveling these complexities may enhance the development of targeted treatments and improve therapeutic outcomes [5].

Differing prevalence rates and key mutation patterns in OPSCC have been reported across various studies, highlighting the heterogeneity of this disease. TP53 mutations, for instance, were found in 15% of recurrent or metastatic HPV-positive OPSCC cases, compared to just 3% in primary HPV-positive HNSCC, as reported by Ashford et al. [10] Similarly, another study analyzing TCGA data found TP53 mutations in only 2% of HPV-positive tumors [11]. PIK3CA mutations showed a prevalence of 20% in a cohort comparing HPV-positive and HPV-negative OPSCC, emphasizing its role as a frequent genetic alteration in these cancers [12], which was similar to our results of 26%. KMT2D mutations were identified as the most frequently mutated gene in one OPSCC cohort, with a predominance of truncating mutations [13]. The key mutations identified in this cohort have direct clinical relevance and therapeutic implications. Alterations in the *PIK3CA* gene, found in 26% of our samples, activate the PI3K-AKT-mTOR pathway and are predictive biomarkers for targeted therapies; inhibitors of this pathway are currently under investigation in multiple clinical trials for HNSCC. Mutations in *TP53* are not only prognostic markers associated with aggressive disease and treatment resistance but may also create vulnerabilities to novel therapies like Wee1 inhibitors or other DNA damage response agents [11]. Furthermore, mutations in the epigenetic modifier *KMT2D* are increasingly recognized as drivers in HNSCC and could represent a future target for therapies aimed at chromatin remodeling, potentially re-sensitizing tumors to standard treatments or immunotherapy [11].

In the NOTCH1 pathway, mutations were found to be more prevalent in recurrent or metastatic HPV-positive HNSCC (16%) compared to primary cases, where they were less frequently observed [12]. Another study reported NOTCH1 mutations in 11% of HNSCC tumors, underscoring their tumor-suppressive role [11]. FAT1 mutations, conversely, were more frequently associated with HPV-negative OPSCC and were particularly prevalent in patients without local or distant recurrence [14]. Finally, TERT promoter mutations (−124 C>T and −146 C>T) were detected in 31.3% of OCSCC patients, where they significantly influenced prognosis [15]. These findings illustrate the variability in mutational prevalence across studies and cohorts, reflecting the complexity of OPSCC biology and the potential for targeted therapies to address its diverse molecular landscape.

Human papillomavirus (HPV) status plays a significant role in influencing mutation profiles and treatment outcomes in oropharyngeal squamous cell carcinoma (OPSCC). HPV-positive OPSCC is characterized by distinct genetic and clinical features compared to HPV-negative OPSCC. HPV-positive tumors often exhibit fewer mutations in the TP53 gene, as HPV oncoproteins E6 and E7 facilitate degradation of p53 and Rb proteins, circumventing the need for TP53 mutations for oncogenesis [14]. HPV-positive OPSCC generally has a better prognosis, responding more favorably to chemoradiation and exhibiting improved overall survival rates compared to HPV-negative cases [16]. However, despite the generally favorable outcomes, a subset of HPV-positive OPSCC patients can exhibit aggressive disease and recurrence, sometimes with unique mutation profiles, such as higher mutation rates in genes like HRAS, PIK3R1, STK11, and TP63, which may contribute to a poorer prognosis [14]. The distinct molecular characteristics of HPV-positive OPSCC, including a lower mutation burden but higher prevalence of epigenetic changes, underpin its unique response to treatment and highlight the importance of HPV status in guiding therapeutic strategies [12]. These findings align closely with the results presented, which detailed the prevalence of key mutations such as TP53, PIK3CA, and KMT2D, as well as their differential enrichment in primary versus metastatic tumors. The observed patterns of co-occurrence, such as between PIK3CA and FBXW7, and mutual exclusivity, such as between TP53 and CYLD, are expanded upon here to underscore their potential roles in driving tumor progression and activating distinct pathways. By linking these results to broader implications for OPSCC biology, the discussion emphasizes how these insights can guide future research and the development of targeted therapeutic strategies.

TP53 mutations are frequently observed in HNSCC and are associated with poor prognosis. They can serve as potential biomarkers for aggressive disease and treatment resistant in HPV-positive OPSCC. These mutations are more prevalent in HPV-negative HNSCC, but their presence in HPV-positive cases can indicate a worse prognosis [16]. Alterations in the PIK3CA gene are common in these carcinomas and are linked to the PI3K-AKT-mTOR pathway, which is in cell growth and survival. These mutations can serve as predictive biomarkers for targeted therapies, with inhibitors showing promise in clinical trials [17]. Concurrent chemoradiotherapy (CCRT) shows promise for unresectable OSCC [18]. For HPV-associated oropharyngeal squamous cell carcinoma, ongoing randomized controlled trials such as RTOG 1016 and De-ESCALaTE are investigating de-escalation treatment protocols, focusing on reducing toxicities while maintaining effectiveness [19]. Additionally, a phase 1 clinical trial targeting HPV-16 E7 demonstrated robust tumor regression in patients through the use of engineered T cells, underscoring the potential for innovative immunotherapies in this patient population [20].

KMT2D mutations are frequently observed in PBV-positive OPSCC and may play a role in tumor recurrence. They are associated with DNA damage repair and could potentially be targeted [14,21]. The presence of genomic alterations such as mutations in the DNA repair pathways suggest the potential for immunotherapy, including checkpoint inhibitors, in treating recurrent/metastatic disease. Biomarkers like ctHPVDNA are emerging as tools for monitoring treatment response and detecting recurrence earlier than imaging [21], which is particularly relevant to our study as it highlights the need for a deeper understanding of the genomic landscape of OPSCC. By identifying key mutations such as TP53, PIK3CA, and KMT2D, and their patterns of enrichment in primary and metastatic tumors, our study contributes to the foundational knowledge required to develop and refine biomarkers like ctHPVDNA. These findings provide valuable insights into tumor-specific genetic alterations that could enhance the sensitivity and specificity of such biomarkers for tracking disease progression and therapeutic response in OPSCC.

Several limitations of this study should be acknowledged. Over 90% of data was missing for specific copy number alterations and structural variants, precluding their inclusion in the analyses and potentially omitting important insights into the genomic landscape of OPSCC. Additionally, survival data was not available for the patient cohort, limiting the ability to assess the clinical outcomes associated with specific mutations. Variability in genomic sequencing pipelines across participating institutions may have introduced inconsistencies in the data, potentially affecting the reliability of the findings. The reliance on the AACR GENIE database, which contains less than 1% of the samples available in The Cancer Genome Atlas, may also limit the generalizability of the results, as it might not fully represent the genomic diversity of OPSCC. Most importantly, the lack of HPV status data prevented the stratification of our cohort into HPV-positive and HPV-negative subgroups, which are known to have fundamentally different genomic landscapes and clinical behaviors; this remains the most significant confounding factor in our analysis. Furthermore, many findings, including gender-specific mutations and differences between primary and metastatic tumors, were associated with high q-values, indicating that these differences may not be statistically significant after correction for multiple comparisons. These limitations underscore the need for further studies with more comprehensive datasets and standardized methodologies to validate and expand upon these findings.

## 5. Conclusions

This genomic analysis of 412 OPSCC tumors identifies critical mutational patterns and delineates key differences between primary and metastatic disease. Our findings confirm the high prevalence of clinically actionable mutations in *TP53* and *PIK3CA* and uncover novel metastatic-enriched genes such as *CBLB* and *BUB1B*, pointing to pathways of immune evasion and genomic instability as potential drivers of progression. The observed patterns of mutational co-occurrence and mutual exclusivity highlight the complex genetic interplay that could be exploited for combination therapies. While limited by the absence of HPV status and clinical outcome data, this study provides a robust foundation for future research. The critical next steps are to validate these genomic signatures in clinically annotated cohorts, stratified by HPV status, and to functionally investigate their role in therapeutic resistance. Ultimately, integrating such detailed genomic profiling into clinical practice holds the promise of guiding personalized treatment strategies and improving survival for patients with oropharyngeal cancer.

## Figures and Tables

**Table 1:** Oropharynx Squamous Cell Patient Demographics.

Patient Demographic	Total (N)	Percentage (%)
Total Patients	401	100.0
Sex		
Male	338	84.3
Female	63	15.7
Age Group		
Adult (≥18)	399	99.5
Pediatric (<18)	1	0.2
Unspecified	1	0.2
Race		
White	337	84.0
Black	15	3.7
Asian	11	2.7
Other	10	2.5
Unspecified	28	7.0
Ethnicity		
Non-Spanish/Non-Hispanic	336	83.8
Spanish/Hispanic	21	5.2
Unspecified	44	11.0
Sample Information	(N = 412 Samples)	(%)
Primary Tumor	216	52.4
Metastasis	181	43.9
Unspecified	15	3.6

**Table 2:** TP53 Mutations in OPSCC.

Type of Mutation	Count	Percentage (%)
Missense	76	61.3
Nonsense	21	16.9
Splice	12	9.7
Frameshift Deletion (FS del)	8	6.5
Frameshift Insertion (FS ins)	4	3.2
In-Frame Deletion (IF del)	2	1.6
In-Frame Insertion (IF ins)	1	0.8

**Table 3:** KMT2D Mutations in OPSCC.

Type of Mutation	Count	Percentage (%)
Missense	43	48.3
Nonsense	30	33.7
FS del	8	9.0
Splice	6	6.7
FS ins	1	1.1
IF del	1	1.1

**Table 4:** Male vs. Female Gene Mutations.

Gene	Cytoband	(A) Female	(B) Male	Log2 Ratio	*p*-Value	Enriched in
ZNF750	17q25.3	0 (0.00%)	4 (36.36%)	<−10	2.57 × 10^−4^	(B) Male
MET	7q31.2	4 (6.25%)	2 (0.58%)	3.43	6.39 × 10^−3^	(A) Female
TP53	17p13.1	26 (40.00%)	83 (23.92%)	0.74	9.15 × 10^−3^	(A) Female
TRAF3	14q32.32	2 (16.67%)	0 (0.00%)	>10	0.0136	(A) Female
EP300	22q13.2	12 (23.08%)	31 (10.37%)	1.15	0.0192	(A) Female
ELANE	19p13.3	2 (20.00%)	0 (0.00%)	>10	0.0204	(A) Female
FGA	4q31.3	0 (0.00%)	1 (50.00%)	<−10	0.0299	(B) Male
POLQ	3q13.33	2 (20.00%)	1 (1.41%)	3.83	0.0389	(A) Female
TGFBR2	3p24.1	3 (11.54%)	4 (2.15%)	2.42	0.0412	(A) Female
DMD	Xp21.2-p21.1	2 (11.76%)	0 (0.00%)	>10	0.043	(A) Female
STAG2	Xq25	4 (7.69%)	6 (2.01%)	1.94	0.0455	(A) Female
ERCC3	2q14.3	3 (6.25%)	3 (1.11%)	2.49	0.0465	(A) Female
MSH2	2p21-p16.3	4 (7.27%)	6 (1.94%)	1.91	0.0482	(A) Female
HIST1H3D	6p22.2	2 (10.00%)	1 (0.79%)	3.67	0.0487	(A) Female
PRCC	1q23.1	1 (100.00%)	0 (0.00%)	>10	0.0526	(A) Female
KRAS	12p12.1	3 (4.62%)	3 (0.86%)	2.42	0.0527	(A) Female
PLCG1	20q12	1 (100.00%)	0 (0.00%)	>10	0.0556	(A) Female
NSD3	8p11.23	3 (15.00%)	5 (3.42%)	2.13	0.0568	(A) Female

Note: *p*-values presented are nominal (uncorrected). Trends observed here should be interpreted as exploratory, as strict False Discovery Rate (FDR) correction may affect statistical significance.

**Table 5:** Primary vs. Metastatic Gene Mutations.

Gene	Cytoband	(A) Metastasis	(B) Primary	Log2 Ratio	*p*-Value	Enriched in
TP53	17p13.1	33 (18.23%)	72 (33.33%)	−0.87	8.75 × 10^−4^	(B) Primary
CBLB	3q13.11	5 (12.20%)	0 (0.00%)	>10	8.85 × 10^−4^	(A) Metastasis
BUB1B	15q15.1	4 (10.53%)	0 (0.00%)	>10	3.03 × 10^−3^	(A) Metastasis
EIF4A2	3q27.3	12 (13.95%)	0 (0.00%)	>10	3.67 × 10^−3^	(A) Metastasis
CDKN2A	9p21.3	11 (6.08%)	30 (13.89%)	−1.19	0.0126	(B) Primary
PTEN	10q23.31	22 (12.15%)	11 (5.09%)	1.26	0.0166	(A) Metastasis
NOTCH2	1p12	4 (2.38%)	16 (8.12%)	−1.77	0.0199	(B) Primary
TSC1	9q34.13	1 (0.58%)	9 (4.39%)	−2.92	0.0245	(B) Primary
APC	5q22.2	3 (1.81%)	14 (6.73%)	−1.9	0.025	(B) Primary
TLR4	9q33.1	3 (18.75%)	1 (1.61%)	3.54	0.0256	(A) Metastasis
BRCA2	13q13.1	5 (2.92%)	17 (8.50%)	−1.54	0.0271	(B) Primary
TGFBR2	3p24.1	1 (0.81%)	5 (6.49%)	−3	0.0324	(B) Primary
KDM6A	Xp11.3	14 (9.15%)	6 (3.24%)	1.5	0.0348	(A) Metastasis
SOX2	3q26.33	14 (9.15%)	6 (3.24%)	1.5	0.0348	(A) Metastasis
RAD54B	8q22.1	3 (15.00%)	0 (0.00%)	>10	0.0369	(A) Metastasis
NOTCH1	9q34.3	17 (9.44%)	36 (16.67%)	−0.82	0.0386	(B) Primary
FOXA1	14q21.1	8 (5.97%)	1 (0.89%)	2.74	0.0423	(A) Metastasis
PIK3CB	3q22.3	7 (5.65%)	0 (0.00%)	>10	0.0458	(A) Metastasis
PRKCI	3q26.2	7 (7.22%)	3 (1.95%)	1.89	0.0488	(A) Metastasis
CYLD	16q12.1	21 (18.58%)	18 (10.47%)	0.83	0.0549	(A) Metastasis

Note: *p*-values presented are nominal (uncorrected). Trends observed here should be interpreted as exploratory, as strict False Discovery Rate (FDR) correction may affect statistical significance.

**Table 6:** Co-occurrence and Mutual Exclusivity of Mutations.

A	B	Neither	A Not B	B Not A	Both	Log2 Odds Ratio	*p*-Value	Tendency
PIK3CA	FBXW7	211	57	10	20	2.888	<0.001	Co-occurrence
KMT2D	FBXW7	229	39	15	15	2.554	<0.001	Co-occurrence
TP53	CYLD	153	56	35	0	<−3	<0.001	Mutual exclusivity
PIK3CA	CYLD	148	61	34	1	<−3	<0.001	Mutual exclusivity
TP53	TERT	206	50	15	15	2.043	<0.001	Co-occurrence
PIK3CA	KMT2D	191	53	30	24	1.528	<0.001	Co-occurrence
TERT	FAT1	159	16	21	10	2.242	0.001	Co-occurrence
NOTCH1	PRKDC	108	17	11	9	2.378	0.002	Co-occurrence
KMT2D	FAT1	157	22	20	11	1.973	0.003	Co-occurrence
NOTCH1	TERT	220	36	19	11	1.823	0.004	Co-occurrence
TP53	FAT1	145	34	17	14	1.812	0.004	Co-occurrence
FAT1	KMT2C	123	11	11	6	2.609	0.005	Co-occurrence
PIK3CA	KMT2C	102	32	7	10	2.187	0.007	Co-occurrence
TP53	NOTCH1	199	52	29	18	1.248	0.014	Co-occurrence
FBXW7	KMT2C	124	10	12	5	2.369	0.015	Co-occurrence
NOTCH1	FAT1	155	24	21	10	1.621	0.015	Co-occurrence
PIK3CA	NOTCH1	193	58	28	19	1.175	0.018	Co-occurrence
TP53	PRKDC	99	26	11	9	1.639	0.026	Co-occurrence
FBXW7	CYLD	184	25	35	0	<−3	0.031	Mutual exclusivity
KMT2D	PRKDC	103	22	12	8	1.642	0.034	Co-occurrence
KMT2D	NOTCH1	211	40	33	14	1.162	0.037	Co-occurrence
PRKDC	CYLD	100	20	23	0	<−3	0.044	Mutual exclusivity
PIK3CA	EP300	203	64	18	13	1.196	0.049	Co-occurrence
TERT	KMT2C	115	15	12	5	1.676	0.058	Co-occurrence

## Data Availability

The data presented in this study are available from the AACR GENIE Database at https://genie.cbioportal.org/ (accessed on 22 July 2024).
